# High-temperature quantum anomalous Hall effect in honeycomb bilayer consisting of Au atoms and single-vacancy graphene

**DOI:** 10.1038/srep16843

**Published:** 2015-11-17

**Authors:** Yan Han, Jian-Guo Wan, Gui-Xian Ge, Feng-Qi Song, Guang-Hou Wang

**Affiliations:** 1 National Laboratory of Solid State Microstructures and Department of Physics, Nanjing University, Nanjing 210093, China; 2 Collaborative Innovation Center of Advanced Microstructures, Nanjing University, Nanjing 210093, China; 3 Key Laboratory of Ecophysics and Department of Physics, College of Scienceence, Shihezi University, Xinjiang 832003, China

## Abstract

The quantum anomalous Hall effect (QAHE) is predicted to be realized at high temperature in a honeycomb bilayer consisting of Au atoms and single-vacancy graphene (Au_2_-SVG) based on the first-principles calculations. We demonstrate that the ferromagnetic state in the Au_2_-SVG can be maintained up to 380 K. The combination of spatial inversion symmetry and the strong SOC introduced by the Au atoms causes a topologically nontrivial band gap as large as 36 meV and a QAHE state with Chern number C = −2. The analysis of the binding energy proved that the honeycomb bilayer is stable and feasible to be fabricated in experiment. The QAHEs in Ta_2_-SVG and other TM_2_-SVGs are also discussed.

Quantum anomalous Hall Effect (QAHE) is getting more and more attention not only because of its Hall conductance quantized into the unit of e^2^/h but also its realization absent of strong magnetic fields. Many theoretical efforts have been made to search suitable material systems to realize the QAHE^1^,^2^,^3^,^4^,^5^,^6^,^7^,^8^. However, no substantial experimental progress was reported until Chang *et al*. for the first time observed the QAHE state in a magnetic topological insulator film composed of chromium-doped (Bi,Sb)_2_Te_3_ in 2013^9^. Nevertheless, the realization temperature of QAHE is rather low, only ~30 mK, which can hardly be utilized for practical purposes. The realization temperature of QAHE is mainly limited by the properties related to the magnetism of the material. In the QAHE, it is the internal magnetization that breaks the time-reversal symmetry. The strength of the ferromagnetic coupling among magnetic particles determines Curie temperature at which the material transforms from ferromagnetic phase to paramagnetic phase, while the strength of spin-orbital coupling (SOC) determines the magnitude of the bulk band gap and the direction of the internal magnetization. The QAHE can be realized only in a two-dimensional system, in which the longitudinal current and Hall voltage are in the plane and perpendicular to the internal magnetization direction. So only the systems with easy magnetization axis perpendicular to the 2D plane and with strong ferromagnetic coupling among magnetic particles are expected for the high-temperature QAHE.

Besides the breaking of time-reversal symmetry caused by the internal magnetization, the spatial inversion symmetry also play an important role in the QAHE. In various Hall effects, the Hall current is attributed to the anomalous velocity, which is always transverse to the electric field and proportional to the Berry curvature of the band^10^,^11^,^12^. The Berry curvature will vanish throughout the Brillouin zone if time-reversal and spatial inversion symmetries simultaneously exist in the crystal^13^. So the anomalous velocity manifests itself only in the system with broken either time-reversal or spatial inversion symmetry such as ferromagnetic crystal. In 1988, Haldane proposed a toy model to realize QAHE^1^. In this model the ions were arranged in a planar, honeycomb lattice to keep the spatial inversion symmetry and a periodic local magnetic-flux density normal to the 2D plane was added to the lattice to break the time-reversal invariance. Today we know that the lattice with the spatial inversion symmetry in the Haldane model is actually as same as that of the graphene.

Graphene is a fantastic 2D material system with extraordinary electrical, magnetic, and mechanical properties^14^,^15^,^16^,^17^,^18^,^19^. Some recent investigations have predicted that the graphene can generate quantum spin Hall effect when the intrinsic spin-orbit coupling (SOC) is considered^20^,^21^. However, due to non-ferromagnetism and weak intrinsic SOC strength, the QAHE can hardly be realized in a perfect graphene. Recently, some groups have proposed adsorbing the transition-metal (TM) atoms on the graphene^22^,^23^,^24^ for magnetizing the graphene and inducing strong Rashba SOC^25^,^26^ to break the time-reversal symmetry. However, it is difficult for such kind of hybrid system to be experimentally realizable due to high mobility of TM atoms on the surface of perfect graphene. Previous experimental and theoretical studies have demonstrated that TM adatoms are active on the surface of prefect graphene and tend to aggregate together to form atomic clusters^27^,^28^,^29^,^30^. Fortunately, graphene with defects e.g. single-vacancy (SV), which can be facilely produced during growth or be deliberately induced by electron or ion irradiation^31^,^32^, was found more attractive to metal atoms than perfect graphene^33^,^34^,^35^. But there is a drawback of introducing SVs into the graphene, i.e. original spatial inversion symmetry of the graphene may be broken by the SVs. If the spatial inversion symmetry is taken into account when the SVs are introduced into the graphene, not only the breaking of the time-reversal symmetry, but also the spatial inversion symmetry may be fulfilled providing a possibility of nonzero Berry curvature throughout the Brillouin zone. So a stable honeycomb hybrid system based on the SV graphene adsorbing TM atoms may provide us a promising avenue to realize the QAHE at high temperature.

In this paper, the high-temperature QAHE is proposed to be realized in a honeycomb bilayer consisting of Au atoms and SV graphene (Au_2_-SVG) based on first-principles calculations. The magnetism of the Au_2_-SVG is first studied, which shows that the easy magnetization axis is perpendicular to the 2D plane and the ferromagnetic state can be maintained up to 380 K. We then illustrate that the combination of the spatial inversion symmetry and the strong SOC causes a topologically nontrivial band gap as large as 36 meV. The Berry curvature is further analyzed to confirm that the Au_2_-SVG bilayer can produce a QAHE state with a nonvanishing Chern number C = -2. The anomalous conductance as a function of Fermi level is also calculated to find a quantized Hall conductance platform signifying the realization of the QAHE. The honeycomb bilayer is proved to be stable and feasible in experiment by the analysis of the binding energy. In addition to the Au_2_-SVG, Ta_2_-SVG is proved to be another potential system to realize the QAHE.

## Results

### Structure and Magnetism

**Figure 1:**
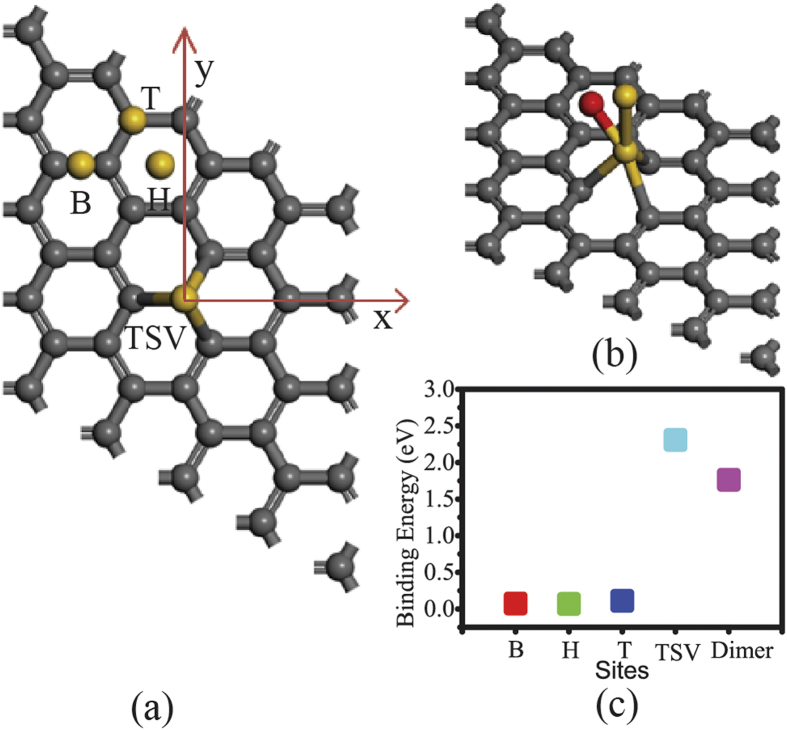
Configurations of Au atoms adsorbed on SV graphene and corresponding binding energy map. (**a**) The schematic diagram of a single Au atom adsorbed at different positions of a 4 × 4 SV graphene supercell. The bridge site (B), hollow site (H), top site (T) and the top of single vacancy site (TSV) are marked as B, H, T, TSV, respectively. We set a coordinate system in the calculations of MAE. x axis and y axis are parallel and perpendicular to the C-C bond, respectively, while z axis is perpendicular to the graphene plane. (**b**) Two lowest-energy configurations with the same binding energies for the Au_2_ dimer adsorbed at TSV of SV graphene. The upper Au atoms of the two configurations are marked in yellow and red, respectively. (**c**) The average binding energy map of the Au atom located at B, H, T, TSV and Au_2_ dimer at TSV.

To find the systems with easy-magnetization axis perpendicular to the 2D plane, we calculated the magnetic anisotropy energy (MAE) for all the hybrid systems composed of a SV graphene adsorbing one 5d-TM atom (TM = Hf, Ta, W, Re, Os, Ir, Pt and Au), in which one 5d TM atom is adsorbed on the top of the SV (TSV) in the graphene as shown in Fig. 1(a). The SVG was modeled as a 4 × 4 graphene supercell in which a carbon atom is removed to introduce a SV defect, as shown in Fig. 1(a). To avoid the interaction between neighboring images, the vertical distance between neighboring layers was set to 15 Å. For simplicity, the hybrid system is denoted as TM-SVG. A 5 × 5 × 1 Γ-centered k-point mesh was used to sample the Brillouin zone of the supercell. After the structure optimizations, the energy differences between two magnetization directions, i.e. Ex–Ey, Ey–Ez and Ez–Ex, were calculated to determine the MAE of the system. In addition, spin-polarized calculations were performed to obtain the magnetic moment of the system.

The calculation results for all the hybrid systems are listed in Table 1. The hybrid systems of Hf-SVG, Os-SVG and Pt-SVG show zero magnetic moment, which are ignored directly for further study. Among the remaining systems, Re-SVG, Ir-SVG and Au-SVG have a magnetic moment of 1 μ_B_, while Ta-SVG and W-SVG have 0.517 μ_B_ and 2 μ_B_, respectively. In a 2D QAHE system, the longitudinal current and Hall voltage are in the plane while perpendicular to internal magnetization direction, so only the systems with easy-magnetization-axis perpendicular to the SVG plane are expected. As listed in Table 1, only three hybrid systems, i.e. Ta-SVG, Re-SVG and Au-SVG, meet this condition. Among them, the Au_2_-SVG has the largest MAE value (21.926 meV), indicative of the strongest SOC and strongest tendency to magnetize spontaneously along the z axis. To clearly expound the QAHE in the honeycomb bilayer, we focus on the hybrid systems consist of Au atoms and the SV graphene.

To verify the stability of the hybrid systems consist of Au atoms and the SV graphene (Au_n_-SVG), we carry out the calculations of the binding energy per atom, which is defined as


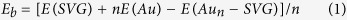


where E(SVG), E(Au), and E(Au_n_-SVG) refer to the energy of SV graphene, Au atom, and the whole system consisting of Au_n_ and SV graphene, respectively. Four highly symmetrical sites, i.e. the top of the SV defect (TSV), the bridge site (B), the hollow site (H) and the top site (T) far away from the SV defect, were considered, as shown in Fig. 1(a). The calculation results show that the binding energy is 2.308 eV, 0.075 eV, 0.07 eV, 0.111 eV for the configuration of Au atom adsorbed at TSV, B site, H site, T site respectively. The binding energies of an Au atom adsorbed on a SV graphene are schematically illustrated in Fig. 1(c). We can see that the E_b_ of Au atom adsorbed at TSV is greatly larger than that of the other three configurations. In addition, the E_b_ value of the Au atom adsorbed at B, H and T site is so small that a very little energy can detach the Au atom from the surface of graphene. If the E_b_ value is roughly deemed as the energy that is needed to detach an Au atom from the graphene, the average life of an Au atom located at TSV, B, H and T site at room temperature (T~300 K) can be easily estimated by using Arrhenius equation τ = (h/k_B_T)exp(E_b_/k_B_T), where k_B_, h, T and E_b_ are Boltzmann constant, Planck constant, temperature and binding energy, respectively. Our calculation show that the average lifetime of Au atom at B, H and T site is less than 1.16 × 10^−11^ s, while the average lifetime of Au atom at TSV site is 8.41 × 10^25^ s. So even if an Au atom is randomly deposited on a SV graphene at site except TSV, it will migrate freely on the graphene due to the very short average life time and be dragged into the TSV and fixed there firmly at last. The analysis of the binding energy shows that the hybrid systems are rather stable.

**Figure 2:**
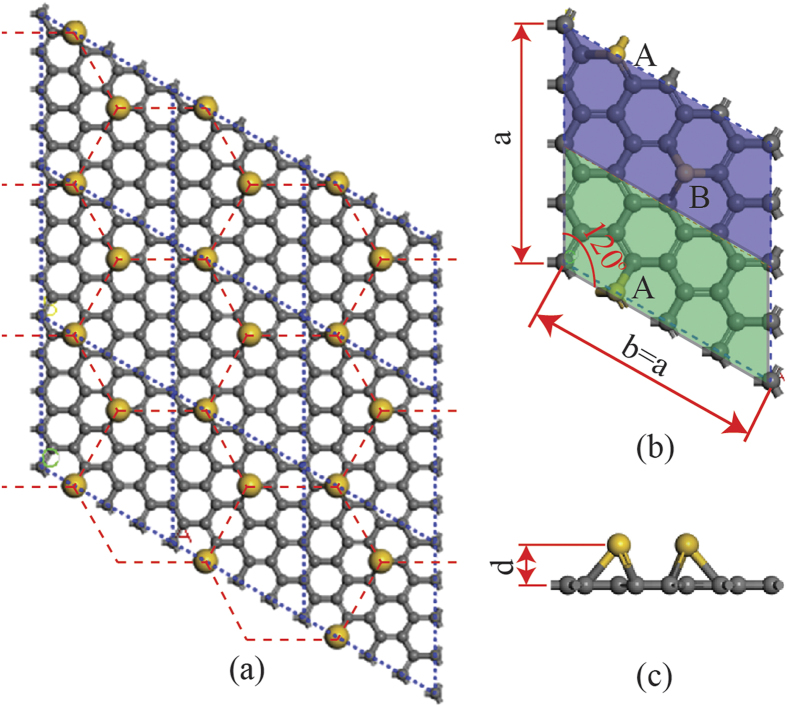
Structural schematic diagram of the bilayer. (**a**) The top view of the Au_2_-SVG bilayer composed of 3 × 3 unit cells. The upper Au atom layer and the lower SVG layer are in the same honeycomb pattern with the C_6ν_ symmetry. The boundaries of all unit cells are marked by blue dashed lines. (**b**,**c**) The top view and side view of an enlarged unit cell, respectively. The unit cell contains sublattice A marked in green and sublattice B marked in blue, in which the SVs (or Au atoms) are at two given positions of the graphene.

Although introducing SVs in graphene can greatly enhance the stability of the TM adatoms on graphene, the original spatial inversion symmetry of the graphene is also broken by the SVs, resulting in the so-called staggered AB sublattice potentials^22^,^36^. If the staggered AB sublattice potentials are larger than the exchange field, it will suppress the joint effect from Rashba SOC and exchange field, leading to a quantum valley Hall phase instead of QAHE^13^,^22^,^36^. Therefore, the SVs introduced into the graphene should be in a pattern with the same spatial inversion symmetry as that of the graphene. Figure 2(a) shows the structural schematic diagram of the hybrid system, in which the unit cell boundaries are shown by blue dashed lines. The arrangement of SVs is in a honeycomb pattern and the Au atoms in the same pattern are adsorbed on the top of the SVs. The whole system has high C_6ν_ symmetry. The details of the unit cell of the system are shown in Fig. 2(b,c). The unit cell (denoted as Au_2_-SVG) is composed of a 4 × 4 supercell of SVG where two Au atoms are adsorbed on the top of the SVs. Each unit cell contains two different sublattices, i.e. sublattice A marked in green and sublattice B marked in blue. In each sublattice there are 15 C atoms and 1 Au atoms. Further structural optimizations for the Au_2_-SVG bilayer gave its lattice constants of a = b = 9.957 Å and the spacing between the two layers of d = 1.612 Å.

**Figure 3:**
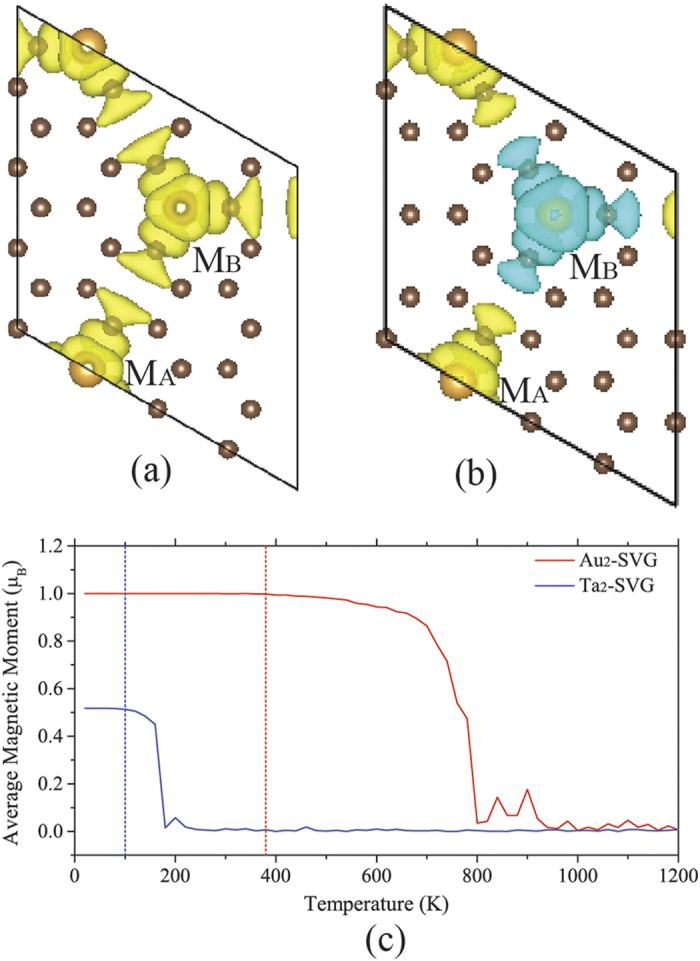
Behavior of ferromagnetic coupling of the bilayer. (**a**) The magnetization charge densities of the ferromagnetic configuration in which the spins of two Au atoms in M_A_ and M_B_ are set as spin-up. (**b**) The magnetization charge densities of the antiferromagnetic configuration in which the spin of two Au atoms in M_A_ and M_B_ are set as spin-up and spin-down, respectively. (**c**) Average magnetic moment as a function of temperature obtained from the Monte Carlo simulation. The Curie temperature is ~380 K for the bilayer Au_2_-SVG and ~100 K for the Ta_2_-SVG bilayer.

The internal spontaneous magnetization is essential for the production of QAHE in the Au_2_-SVG bilayer. To explore the magnetic states of the Au_2_-SVG bilayer, we estimate the strength of exchange coupling between the sublattices by calculating the total-energy difference between spin-antiparallel (antiferromagnetic coupling, AFM) and spin-parallel (ferromagnetic coupling, FM) configurations as follows:


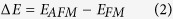


where E_AFM_ and E_FM_ refer to the energy of the spin-antiparallel configuration and spin-parallel configuration respectively. So a positive vale of ΔE means a ferromagnetic state. The magnetization charge densities (spin-up charge density minus spin-down charge density) of FM and AFM configurations are plotted in Fig. 3(a,b). For each Au atom in sublattice A or B, its spin direction always keeps the same as that of the C atoms around it, indicative of the ferromagnetic coupling between the Au atom and the C atoms around it. So both sublattices can be deemed as a magnetic entity (denoted as M_A_ or M_B_ which has a magnetic moment of 1 μ_B_ according to the calculation results listed in Table 1). Evidently, the strength of exchange coupling between M_A_ and M_B_ will determine the magnetic state of the whole system. Our calculations showed that the Au_2_-SVG bilayer is ferromagnetic when it is on the magnetic ground state (ΔE = 35.6 meV).

In experiment, the QAHE can only be realized below Curie temperature Tc^9^. So a high Tc facilitates the realization of QAHE. In order to estimate the Curie temperature where the transition from FM phase to paramagnetic phase occurs in the bilayer, we then performed Monte Carlo simulations using Metropolis algorithm^37^,^38^ in the Ising model, which was used historiclly to study magnetic phase trnsitions. In the calculations, 100 × 100 sublattices were used as the Monte Carlo sampling region. The temperatures specified for Monte Carlo simulations were taken from 20 K to 1200 K with a step length of 20 K. At each temperature, 1 × 10^9^ steps Monte Carlo simulation were implemented and the average magnetic moment was calculated by counting the last 1 × 10^4^ steps simulations. The results are plotted in Fig. 3(c). It is found that the magnetic moment of the system always keeps 1.0 μB in the range from 0 to ~380 K and then starts to drop, indicative of the Curie temperature Tc ~380 K.

The reasonability of this Curie temperature we obtained above was further veryfied by using the mean-field approximation (MFA) method, which obtain a close value of 412 K. In the the classical Heisenberg model, the Hamiltonian is


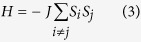


where S_i_ is the spin value of M_A_ or M_B_, J is the exchange interaction strength. The equation (3) can be rewritten as H = −JzS_A_S_B_ assuuming the coordination number of each magnetic entity is z. In the present system, the coordination number is 3 and the spin values of M_A_ and M_B_ are equal, i.e. S_A_ = S_B_ = S = 1/2. Combinding equation (3) and equation (2), we got J = ΔE/(2zS^2^) = 23.7 meV. Within the mean-field approximation (MFA) the thermal behavior is determined by the Brillouin function expression, and the Curie temperature can be estimated as 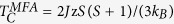. Accordingly, we obtained the Curie temperature Tc = 412 K for the Au_2_-SVGr bilayer, which is close to the value estimated by the Monte Carlo simulations (~380 K).

### Band structures

**Figure 4:**
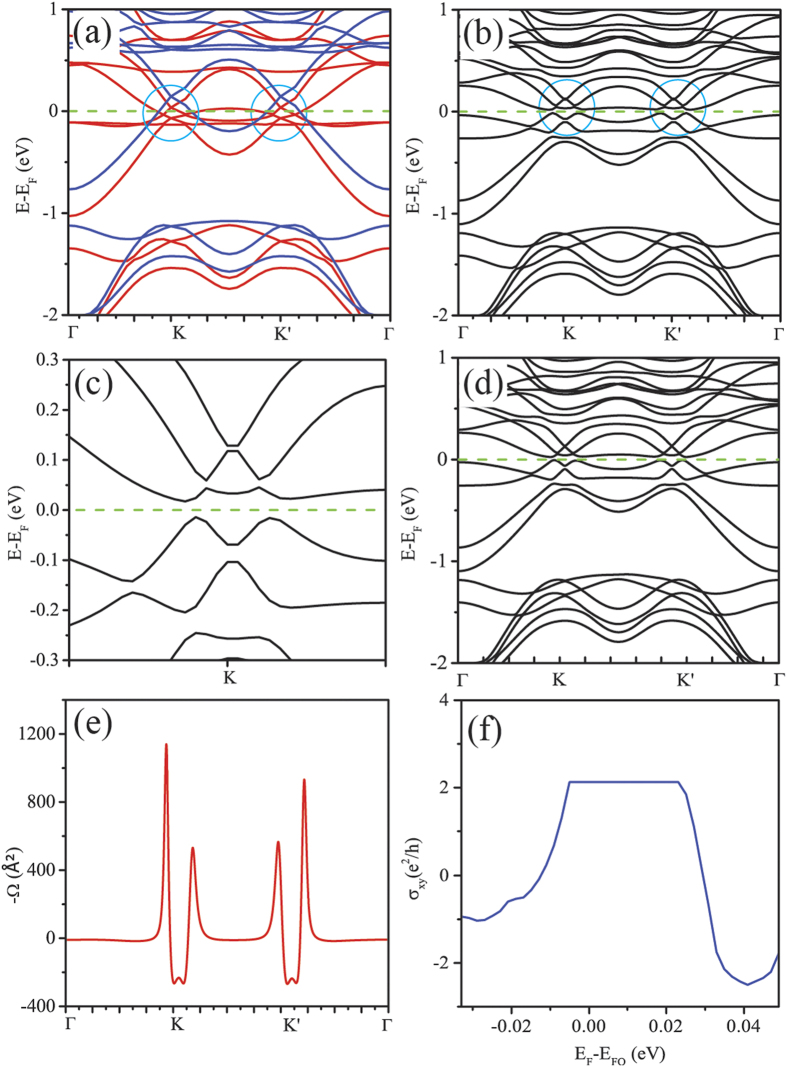
Transport properties of the Au_2_-SVG. (**a**) The band structures including the spin polarization for the Au_2_-SVG bilayer. The spin-up and spin-down bands are marked in red and blue, respectively, and the Fermi level is marked in green dashed line. The spin-up (red) and spin-down (blue) bands intersect forming four crossing points around either K or K’ point marked by circle. (**b**) The band structure including both spin polarization and SOC for the Au_2_-SVG bilayer. A sizable bulk band gap about 36 meV is opened at K and K’ points. (**c**) The magnification of the band gap at K point. (**d**) The band structures of Au_2_-SVG obtained from the Wannier interpolation scheme. (**e**) The negative Berry curvatures Ω(k) of the Au_2_-SVG bilayer along the high symmetry lines. (**f**) The anomalous Hall conductance σ_xy_ as a function of Fermi level for Au_2_-SVG. E_F_ and E_FO_ denote the Fermi level set in the calculation and the original Fermi level respectively.

We subsequently calculated the band structures of the Au_2_-SVG bilayer. Figure 4(a) plots the band structure including spin polarization. The spin-up and spin-down bands intersect at 4 points around either K or K’ point, those are marked by the circles in Fig. 4(a). Among the 4 crossing points, two locate at the Fermi level and another two are above the Fermi level. The band structure including both spin polarization and SOC was further calculated, as shown in Fig. 4(b). A sizable bulk band gap of about 36 meV is opened at K and K’ points in the presence of SOC, while the Fermi level just lies in the gap. The band gap at K point is magnified to be observed clearly as shown in Fig. 4(c). The above calculation results suggest that the Au_2_-SVG bilayer is an ideal candidate for realizing the QAHE. It is known that the degeneracy points (8 crossing points) correspond to monopoles and act as sources and drains of the Berry curvature flux in the momentum space. When the Fermi level lies in a gap introduced by SOC, the Hall conductance will be quantized into integer times of the unit of e^2^/h and will be guaranteed by the gap remaining unchanged after thermodynamic averaging. So the whole system is promising to get a nonzero Chern number C, which indicates a quantized hall conductance of Ce^2^/h, by integrating the Berry curvature over the Brillouin zone. Roughly estimated by E = k_B_T, the large band gap of 36 meV can protect the QAHE state up to 417 K.

### Berry curvature, Chern number, and anomalous hall conductance

The Berry phase analysis in the momentum space^13^ was carried out in order to further identify the nontrivial topological properties of the Au_2_-SVG bilayer. By constructing the Bloch wave functions ψ from the self-consistent potentials, the Berry curvature Ω(k) can be obtained using the following formula^11^,^39^,^40^:


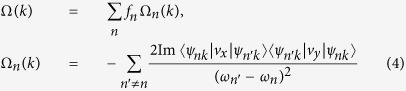


where the summation is over all n valence bands below the bulk band gap, f_n_ is the Fermi-Dirac distribution function, 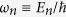, and v_x(y)_ is the velocity operator. In our calculations, the Berry curvatures are calculated by Wannier interpolation through the gauge transfer between the Bloch functions and real space Wannier functions using WANNIER90^41^,^42^. The reliability of the results obtained by the WANNIER90 is verified by the band structure calculated by the WANNIER90 as shown in Fig. 4(d). The band structures of Au_2_-SVG obtained from the Wannier interpolation scheme are the same as that from DFT. The negative Berry curvatures Ω(k) of Au_2_-SVG along the high symmetry lines are plotted in Fig. 4(e). One observes that the nonzero Berry curvatures mainly distribute around the K and K’ points and correspond to the crossing points of spin-up and spin-down bands. There are four distinct positive peaks caused by the four crossing points at the Fermi level. In addition, two small negative peaks appear due to the crossing points above the Fermi level. The Chern number can be obtained by integrating the Berry curvatures Ω over the first Brillouin zone using Eq. (5)^39^,^43^,


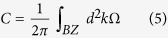


Our data show C = −2 for the insulating state of the Au_2_-SVG bilayer. The nonvanishing Chern number C in the absence of magnetic field distinctly signifies the existence of QAHE state. The anomalous Hall conductance as a function of Fermi level calculated by WANNIER90 is shown in Fig. 4(f). The anomalous conductance σ_xy_ shows a quantized Hall conductance platform with the width of 32 meV, which is very close to the band gap calculated by DFT. The consistency between the width of the Hall conductance platform obtained from WANNIER90 and the band gap from DFT prove the reliability of our calculations again. When the Fermi level lies in the band gap, the anomalous Hall conductance is quantized as 2e^2^/h. When the Fermi level is shifted beyond the platform, quantized platform disappears while the anomalous σ_xy_ remains nonzero value. This can be attributed to the metallic bands when the Fermi level is outside the band gap opened by SOC.

### The feasibility of fabricating the Au_2_-SVG bilayer in experiment

We now illustrate the feasibility of fabricating the Au_2_-SVG bilayer in experiment through a self-assembly process. The honeycomb pattern of SV points in the graphene can be produced precisely using various kinds of technologies^34^. To achieve the ordered deposition of Au atoms onto the surface of the SV graphene, we may control them to deposit one by one. Our calculation results show that the binding energy of the configuration of Au atom adsorbed at the TSV of graphene is as large as 2.308 eV, much larger than those of the other sites, e.g. 0.075 eV for the bridge site (B), 0.07 eV for the hollow site (H) and 0.111 eV for the top site (T). In other words, the first depositing Au atom has to occupy one of SV points in the graphene. To determine the occupying state for the second Au atom, the lowest-energy configuration of Au_2_ dimer at TSV and its binding energy were also calculated. The calculation results show that the Au_2_ dimer at TSV has two lowest-energy configurations with the same binding energy E_b_ = 1.764 eV/atom, as shown in Fig. 1(b). From Fig. 1(c), we can clearly observe that the E_b_ value of the Au_2_ dimer adsorbed at TSV is lower than that of a single Au atom adsorbed at TSV (2.308 eV/atom). Therefore, the second Au atom tends to occupy another SV point rather than combine with the first one to form an Au_2_ dimer. The above analysis of the binding energy proved that it is feasible to fabricate a honeycomb bilayer assembled by Au atoms and SVG layer.

## Discussions

**Figure 5:**
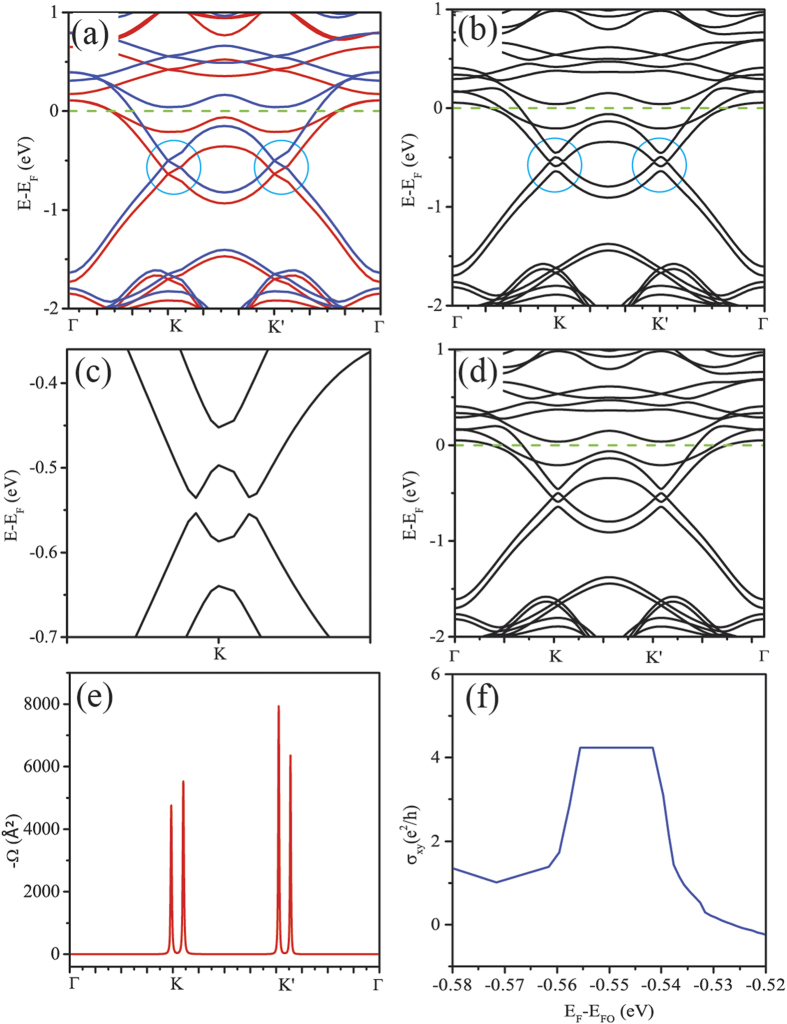
Transport properties of the Ta_2_-SVG. (**a**) The band structures including the spin polarization for the Ta_2_-SVG bilayer. The spin-up and spin-down bands are marked in red and blue, respectively, and the Fermi level is marked in green dashed line. (**b**) The band structure including both spin polarization and SOC for the Ta_2_-SVG bilayer. A sizable bulk band gap about 16 meV is opened at K and K’ points. (**c**) The magnification of the band gap at K point. (**d**) The band structures of Ta_2_-SVG obtained from the Wannier interpolation scheme. (**e**) The negative Berry curvatures Ω(k) of the Ta_2_-SVG bilayer along the high symmetry lines. (**f**) The anomalous Hall conductance σ_xy_ as a function of Fermi level for Ta_2_-SVG. E_F_ and E_FO_ denote the Fermi level set in the calculation and the original Fermi level respectively.

In the Au_2_-SVG bilayer we present here, the key point of realization of QAHE lies in the combination of the spatial inversion symmetry of the whole system and the breaking of the time-reversal symmetry broken by the strong Rashba SOC and magnetism induced by Au atoms and SVs. In the Au_2_-SVG bilayer, the SV (or Au atom) in each A or B sublattice is deliberately arranged at given positions making the whole system maintain a perfect spatial inversion symmetry. We thereby suggest such kind of bilayer structure may be universal to realize the QAHE if the Au atoms are replaced by the other 5d-TM atoms. Noticing that the hybrid system of Ta and SVG has a large MAE with easy magnetization-axis perpendicular to the SVG plane, as listed in Table 1, we constructed a Ta_2_-SVG bilayer using the structure configuration the same as the Au_2_-SVG bilayer. After structural optimizations, we also obtained a stable Ta_2_-SVG bilayer with the lattice constants of a = b = 10.053 Å and layer spacing d = 1.368 Å. The subsequent calculations of band structures and Berry curvatures confirmed that it can also produce a QAHE state. From the band structures shown in Fig. 5(a), one observes that the spin-up and spin-down bands intersect at two points around either K or K’ when only spin polarization is included. A bulk band gap of about 16 meV is opened at K and K’ points after the SOC is considered as shown in Fig. 5(b). The magnification of the band gap at K point is plotted in Fig. 5(c). Since all of the crossing points are below the Fermi level, we tuned the Fermi level into the band gap and then calculated the Berry curvature Ω. As shown in Fig. 5(e), the Berry curvatures Ω around the K point share the same sign as that near the K’ point, meanwhile, the four peaks exactly correspond to the four crossing points in band structure, indicating a nonzero Chern number. By integrating the Berry curvature Ω over the first Brillouin zone using Eq. (5), the Chern number C = −4 is obtained, which indicates four chiral edge channels will appear on each side of the sample. The quantized anomalous Hall conductance platform is also found in the anomalous Hall conductance curve, as shown in Fig. 5(f). The band structure including SOC is also calculated by the WANNIER90 to verify the reliability of the calculation for the Berry curvature. As is shown in Fig. 5(d), the band structures of Ta_2_-SVG obtained from the Wannier interpolation scheme are the same as that from DFT. The consistency between the band structures calculated by WANNIER90 and DFT justify the reliability of the Wannier functions we obtained. Further calculation of ferromagnetic coupling shown in Fig. 2(c) confirms that the QAHE state of the Ta_2_-SVG bilayer can maintain up to ~100 K. Except for the Ta_2_-SVG, our roughly preliminary studies show that some 3d-TM and 4d-TM elements such as V, Fe, Cu, Zn and Nb may be also potential candidates for constructing such kind of bilayer with QAHE, though the band gaps opened in the presence of SOC is not as large as that of the 5d TM_2_-SVG bilayer. In addition, we suggest that using appropriate TM dimers or clusters to replace the TM atom in the TM_2_-SVG bilayer may be another potential avenue.

Although we have demonstrated that the QAHE can be realized in some TM_2_-SVG bilayer, we must recognize that not all the TM_2_-SVG can be used to realize the QAHE. The combination of the spatial inversion symmetry and the breaking of the time-reversal symmetry just provide facilities for the QAHE instead of sufficient conditions. A counter example is the Re_2_-SVG. Our study shows that the SOC in the Re_2_-SVG cannot open a global band gap for the QAHE.

Finally, we will discuss a similar structure to the bilayer Au_2_-SVG we present here. In the Au_2_-SVG bilayer we presented, the two Au atoms are adsorbed at the same side of the graphene. There is a possibility that one Au atom located at A site on one side and another Au atom located at B site on the opposite side as shown in the Supplementary Information. Our calculation results show that the energy of the configuration that the two Au atoms stay on the different side is 0.575 eV lower than that the two Au atoms stay on the same side. But the structure of the Au_2_-SVG is deformed dramatically by the difference of the positions of the Au atoms in z direction as shown in Fig. S1(a) and Fig. S1(b) in the Supplementary Information. The deformed ion structure will affect the electron structure. Our study shows that the SOC cannot open a global band gap in the band structure. The detailed description can be seen in the supplementary Information. So the platform will not appear in the Hall conductance curve, i.e. there is no Hall conductance quantization.

In fact, the SV graphene is placed on the substrate such as SiO_2_ in expement. There is only one side expose to the Au atoms. Once the Au atoms are adsorbed to the SV graphene, it is hard for them to escape from the vacancies due to the strong interaction between the Au atoms and the vacancies. Although the configuration that the Au atoms locate at different sides of the graphene have a slightly lower energy than that the Au atoms locate at the same side, the forming of the configuration can be completely avoided.

In summary, we have predicted the realization of high-temperature QAHE in a honeycomb bilayer containing Au atoms and SV graphene. The easy magnetization axis of the Au_2_-SVG is perpendicular to the 2D plane and the ferromagnetic state can be maintain up to ~380 K. The combination of spatial inversion symmetry and strong SOC introduced by the Au atoms causes a topologically nontrivial band gap as large as 36 meV. The Hall conductance is proved to be quantized as 2e^2^/h by the Berry curvature analysis. The binding energy analysis shows that the bilayer is rather stable and can be achieved in experiment. We also demonstrate that the bilayer Ta_2_-SVG is another potential system to realize the QAHE. Our present work points out a promising avenue to realize the QAHE at high temperature and may stimulate relative experimental progress.

## Methods

The calculations have been performed by the Vienna ab initio simulation package (VASP)^44^ using the projector augmented wave (PAW)^45^,^46^. The exchange and correlation functional are described by the Perdew, Burke, and Ernzerhof (PBE) functional within the generalized gradient approximation (GGA)^47^. The SOC are calculated in a noncollinear mode in VASP^48^,^49^. In the calculations, the cut-off energy for plane wave basis set is 400 eV. The electronic self-consistency loop will be broken when the changes of both the total energy and the band structure energy between two steps are smaller than 10^−4^ eV. The ionic relaxation loop will be broken when all forces are smaller than 10^−2^ eV/Å. In the calculation of magnetic anisotropy energy (MAE) and band structure, the electronic self-consistency loop will be broken when the change in total energy between successive iteration steps are smaller than 10^−7^ eV^50^.

## Additional Information

**How to cite this article**: Han, Y. *et al*. High-temperature quantum anomalous Hall effect in honeycomb bilayer consisting of Au atoms and single-vacancy graphene. *Sci. Rep*. **5**, 16843; doi: 10.1038/srep16843 (2015).

## Supplementary Material

Supplementary Information

## Figures and Tables

**Table 1:** The magnetic moment (Mag) and MAE for the TM-SVG hybrid system (TM = Hf, Ta, W, Re, Os, Ir, Pt and Au).

	Mag (μ_B_)	MAE (meV)			Easy axis
		E_x_-E_y_	E_y_-E_z_	E_z_-E_x_	
Hf-SVG	0	—	—	—	
Ta-SVG	0.517	0.813	7.616	−8.429	z
W-SVG	2	0	−12.868	12.868	x, y
Re-SVG	1	1.774	17.141	−18.915	z
Os-SVG	0	—	—	—	
Ir-SVG	1	0	−1.417	1.417	x, y
Pt-SVG	0	—	—	—	
Au-SVG	1	3.526	18.4	−21.926	z
