# Supplementary material for: High-temperature quantum anomalous Hall effect in honeycomb bilayer consisting of Au atoms and single-vacancy graphene
Source: Sci Rep. 2015 Nov 17;5:16843. doi: 10.1038/srep16843 (PMC4647114; doi:10.1038/srep16843)
Supplement: Supplementary Information [file srep16843-s1.pdf]

Supplementary Information for

High-temperature quantum anomalous Hall effect in honeycomb bilayer consisting of Au atoms and single-vacancy graphene

Yan Han[1,2], Jian-Guo Wan[1,2,*], Gui-Xian Ge[1,3] Feng-Qi Song[1,2]
& Guang-Hou Wang[1,2]

[1] National Laboratory of Solid State Microstructures and Department of Physics, Nanjing University, Nanjing 210093, China

[2] Collaborative Innovation Center of Advanced Microstructures, Nanjing University, Nanjing 210093, China

[3] Key Laboratory of Ecophysics and Department of Physics, College of Scienceence, Shihezi University, Xinjiang 832003, China

*Correspondence and requests for materials should be addressed to Jian-Guo Wan (email: wanjg@nju.edu.cn)

In addition to the Au_2-SVG bilayer we presented in this paper, there is a another possibility that one Au atom located at A site on one side and another Au atom located at B site on the opposite side of the graphene as shown Fig. S1(a) and Fig. S1(b). The structural optimizations show that the lattice constants is 9.947 Å, which is very close to the situation that the two Au atoms stay on the same side of the graphene plane (9.957 Å). The Au atoms are 1.771 Å far away from the graphene plane, which is larger than that the Au atoms stay on the the same side of the graphene (1.612 Å). Our calculation results show that the energy of the configuration that the two Au atoms stay on the different side is 0.575 eV lower than that the two Au atoms stay on the same side. But the structure of the Au_2-SVG is deformed dramatically by the difference of the positions of the Au atoms in z direction as shown in Fig. S1(a) and Fig. S1(b). The C atoms are no longer in the regular hexagons as shown in Fig. S1(a). The amplitude that the C atoms fluctuate along the z direction is about 0.948 Å as shown in Fig. S1(b). The deformed ion structure will affect the electron structure. The corresponding band structures with and without SOC of the system are calculated. The results are shown Fig. S1(c) and Fig. S1(d). We can see that the spin-up band (red) and the spin-down (blue) band cross at two points at the Fermi level and there are only spin-up bands between the two crossing points as shown in Fig. S1(c). When the SOC is included, two local band gaps are open at the two crossing points as shown in Fig. S1(d). Due to the presence of the spin-up bands between the two crossing points, the SOC cannot open a global band gap. So the platform will not appear in the Hall conductance curve, i.e. there is no Hall conductance quantization.

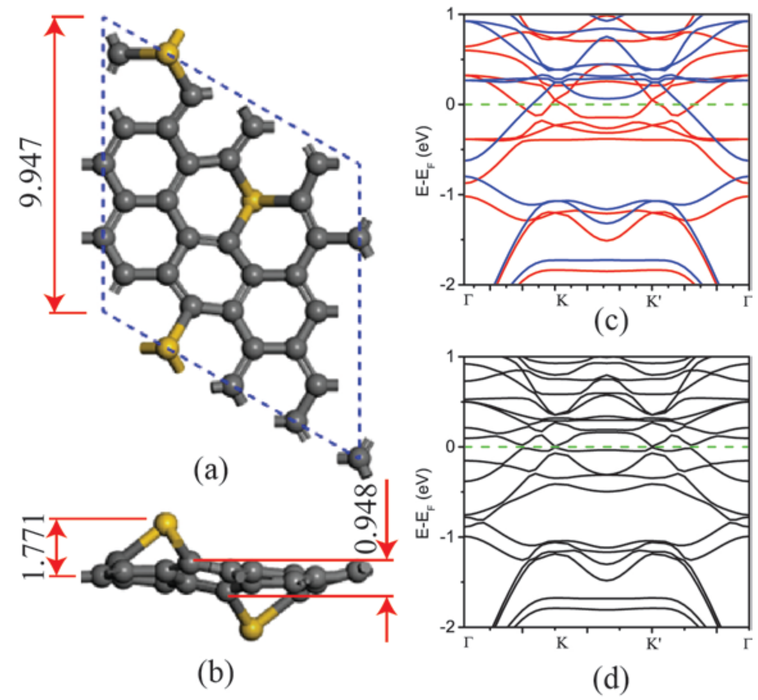

Fig. S1. (a) and (b) The top view and side view of the configuration that the two Au atoms located on the different side of the graphene. The unit of the length is Å. (c) The band structure including only spin polarization. The Spin-up and spin-down bands are marked by red and blue lines respectively. The dashed green line denotes the Fermi level. (d) The band structure including both spin polarization and spin-orbital coupling (SOC).
